# Depression in Adolescents with Asperger’s Syndrome: Long-Term Outcome

**DOI:** 10.1192/j.eurpsy.2022.697

**Published:** 2022-09-01

**Authors:** I. Nicolau, C. Voinea, L. Mateescu

**Affiliations:** 1 “Prof.Dr. Al. Obregia” Psychiatry Clinical Hospital, Child And Adolescent Psychiatry Department, Bucharest, Romania; 2 Carol Davila University of Medicine and Pharmacy, Child And Adolescent Psychiatry Department, Bucharest, Romania

**Keywords:** adolescence, Asperger, Depression

## Abstract

**Introduction:**

Asperger Syndrome (AS) exhibits a particular set of difficulties, the foremost of them being the impairment in social interaction. Furthermore, adolescence by itself is a period burdened with many social challenges and distress. The attempt to adjust to them while having AS is much harder, thus it may constitute the premise for the onset of internalizing symptomatology, in particular depression.

**Objectives:**

This study aimed to observe what factors either improved or hindered the long-term outcome. It was analyzed how the different types of interventions influenced the outcome of these individuals in terms of academic performance, social functioning, psychiatric relapses and quality of life.

**Methods:**

Our lot was represented by 16 patients diagnosed with Asperger Syndrome and Major Depressive Disorder (MDD), followed up on a period between 1 to 10 years. The lot was divided into two groups, each with a different therapeutic plan. The main instruments used were clinical observation and parents’ assessments.

**Results:**

In the first group, the adolescents, treated with a complex intervention which included also a psychotherapeutic component, were found to have a positive outcome, 71,42% of them having no other psychiatric comorbidity than Major Depressive Disorder. The adolescents in the second group, who were treated only with pharmacological treatment, were found to have a negative outcome.

**Conclusions:**

The factors that were found to have the most important impact on the long-term outcome were: the integration in a psychotherapy programme, having family support, compliance with the pharmacological treatment and having MDD as the only psychiatric comorbidity.

**Disclosure:**

No significant relationships.

